# Extended Temperature Range of the Ice-Binding Protein Activity

**DOI:** 10.1021/acs.langmuir.3c03710

**Published:** 2024-03-25

**Authors:** Vera Sirotinskaya, Maya Bar Dolev, Victor Yashunsky, Liat Bahari, Ido Braslavsky

**Affiliations:** † Institute of Biochemistry, Food Science, and Nutrition, Robert H. Smith Faculty of Agriculture, Food and Environment, The Hebrew University of Jerusalem, Rehovot 7610001, Israel; ‡ Faculty of Biotechnology and Food Engineering, Technion, Haifa 3200003, Israel; § The Swiss Institute for Dryland Environmental and Energy Research, Ben Gurion University, Beer-Sheva 84105, Israel

## Abstract

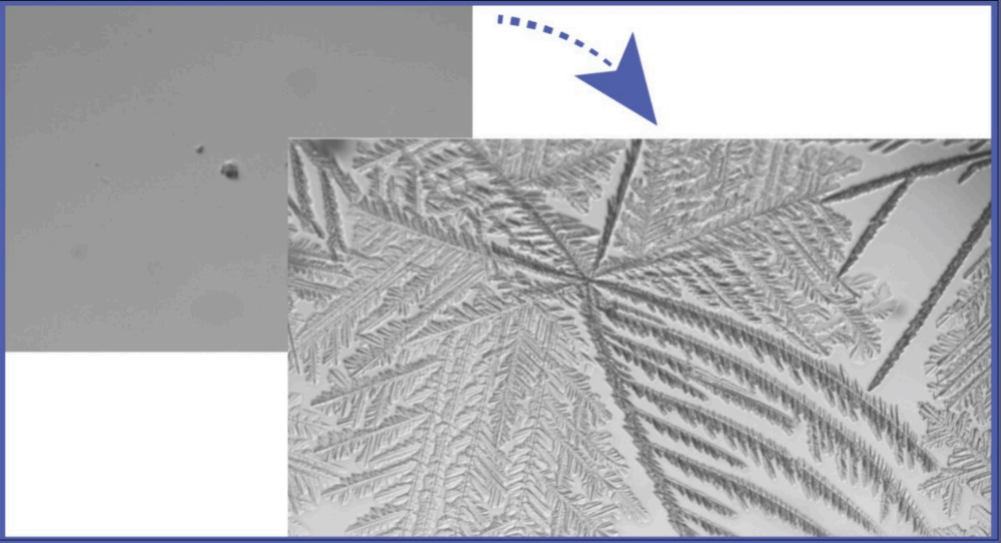

Ice-binding proteins (IBPs) are expressed in various organisms for several functions, such as protecting them from freezing and freeze injuries. Via adsorption on ice surfaces, IBPs depress ice growth and recrystallization and affect nucleation and ice shaping. IBPs have shown promise in mitigating ice growth under moderate supercooling conditions, but their functionality under cryogenic conditions has been less explored. In this study, we investigate the impact of two types of antifreeze proteins (AFPs): type III AFP from fish and a hyperactive AFP from an insect, the *Tenebrio molitor* AFP, in vitrified dimethylsulfoxide (DMSO) solutions. We report that these AFPs depress devitrification at −80 °C. Furthermore, in cases where devitrification does occur, AFPs depress ice recrystallization during the warming stage. The data directly demonstrate that AFPs are active at temperatures below the regime of homogeneous nucleation. This research paves the way for exploring AFPs as potential enhancers of cryopreservation techniques, minimizing ice-growth-related damage, and promoting advancements in this vital field.

## Introduction

Cryopreservation is currently the main method for the long-term storage of cells and tissues. At extremely low temperatures, the diffusion is slow, and molecules do not have enough energy to pass energy barriers for chemical reactions. Therefore, biological activity practically ceases, and the cells and tissues can be preserved. However, ice growth during the cooling and warming stages poses a significant challenge. Intracellular freezing is usually considered to be lethal. Extracellular ice growth leads to water depletion from the solutions, resulting in an elevated solute concentration and diffusion of water out of the cells. This leads to osmotic stress due to heightened intracellular solute concentration, membrane injuries, and physical stress on shrinking cells.^1−3^ Ice recrystallization (IR), the process of enlargement of ice crystals at the expense of smaller crystals, is considered damaging and occurs during the freezing and thawing. The amount of ice and its growth pattern are contingent on the solutes and on the temperature profile through freezing, storage, and thawing.

**Figure 1:**
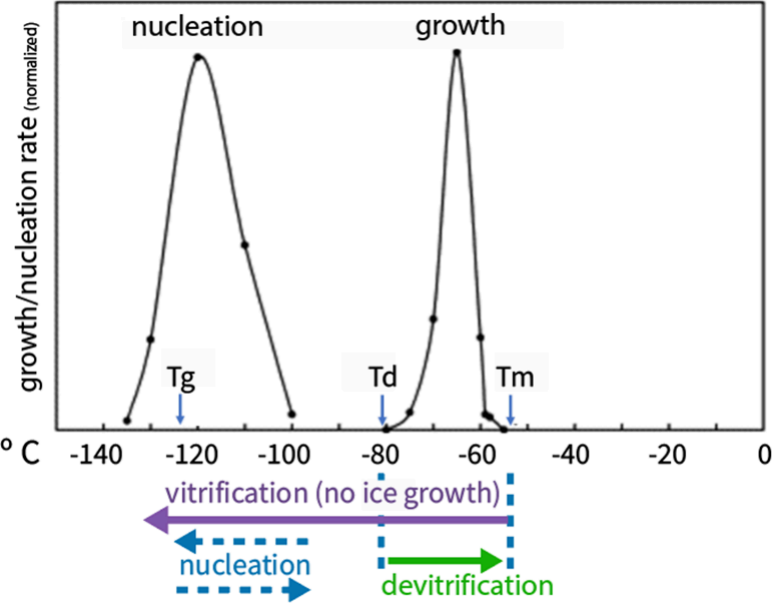
Schematic representation of the effect of temperature on ice nucleation and growth rate during cooling and warming of solutions. Ice nucleation peaks slightly above Tg, and ice growth occurs above Td (the temperature at which devitrification starts during warming), and below Tm., is dependent on CPA concentration. In dilute solutions, Td is closer to Tg; in concentrated solutions, Td is closer to Tm. Dilute CPA solutions show wider and overlapping nucleation and growth curves. The *Y*-axis represents the results of growth rates (for the growth regime) or nucleation rates (for the nucleation regime), normalized from DSC studies. [Modified with permission from ref (5). Copyright 2010, Elsevier.]

The primary approach for mitigating ice growth damage in cryopreservation is through vitrification. Vitrification is the conversion of a liquid to an amorphous solid glass without undergoing crystallization.^4^ This process occurs through rapid cooling, effectively bypassing the ice growth and nucleation zones between the melting temperature (Tm) and the glass-transition temperature (Tg) (see Figure 1). The liquid water molecules do not have sufficient time to organize into a crystalline structure and rigidify into a glass state with exceptionally high viscosity (>10^12^ Pa s).^5^

When the target is much larger than a single cell, it is impractical to obtain stable vitrification solely by fast cooling and heating. Vitrification of biological samples involves a combination of rapid cooling and heating rates, in addition to adding cryoprotective agents (CPAs).^6,7^ CPAs depress the melting temperature (Tm) and the homogeneous nucleation temperature (Th) while also elevating the Tg in a concentration-dependent manner.^8,9^ This results in a narrower temperature difference between Tm and Tg, effectively reducing the ice growth and nucleation phases and enabling vitrification at slower cooling rates.^10^

Bypassing the ice growth and nucleation regime during warming presents a greater challenge compared to cooling. Ice nuclei formed during the cooling process do not grow significantly since at lower temperatures the growth rate is low. Conversely, during the warming phase, these ice nuclei, alongside others formed during the warming phase, undergo growth as the temperature increases (depicted in Figure 1). This phenomenon, termed *devitrification*, is followed by IR, leading to ice-related injuries of the thawed specimens.

To circumvent devitrification, exceedingly high heating rates are essential,^10^ depending on the CPA used. For dimethyl sulfoxide (DMSO), a prevalent CPA in contemporary cryopreservation, warming rates must exceed cooling rates by several orders of magnitude.^9,11^ Achieving such rates is impractical due to complications related to heat transfer.^12^ An alternative approach involves utilizing high concentrations of CPA. Such concentrations (molar range) can lead to osmotic injuries and cytotoxic effects.^13,14^ Furthermore, DMSO may modify protein structure and denaturation,^15^ and, as a cell differentiation inducer, it may influence gene regulation.^16,17^ The apparent toxic effects of high DMSO concentrations drive the exploration of less-toxic alternatives.

One such approach to mitigate devitrification involves the introduction of various ice-active substances. Ice-binding proteins (IBPs), as suggested by their name, possess an inherent capability to bind to ice crystals and nuclei, aiding organisms in surviving freezing conditions.^18^ Through direct interaction with water molecules on the ice surface or at the ice–water interface, IBPs exert significant physical effects on the subsequent growth of the bound ice crystal. IBPs depress the freezing point of an ice crystal in a noncolligative manner by blocking the access of water molecules to the ice surface, resulting in a lower freezing point than the melting point within an IBP solution. This mode of ice growth inhibition markedly differs from the colligative effect of small molecule CPAs used in vitrification. Moreover, IBPs exhibit robust IR inhibition activities.^18^

Studies on IBPs typically concentrate on temperatures ranging from 0 °C to −20 °C, where IR is prominent and IBPs are biologically active. A few recent studies on ice nucleation proved that IBPs^19−22^ and other ice-binding substances^23^ are active at temperatures in the range of homogeneous nucleation. At the conditions studied, IBPs increased the ice nucleation temperature from −38 °C to −34 °C at an IBP concentration of 100 μM.^19−23^ Still, little is known about their functionality under cryogenic conditions.

Several studies have reported predominantly favorable post-thaw cryopreservation outcomes, including vitrified biological samples, with the addition of IBPs.^24−37^ Hey et al. investigated the influence of type I AFP on ice growth velocity in glycerol solution down to −65 °C and did not find a significant effect, even at high protein concentrations. The authors mentioned some effects on the cracking of the vitrified solution but without documentation of the phenomenon.^38^ This finding contrasts Sutton and Pegg’s measurements that presented a shift in DSC thermograms of a cryoprotectant (butan-2,3-diol) when AFP I was added, indicating the elevation of the devitrification temperature.^39^ In another study, a change detected in DSC thermograms in the presence of AFP I was referred to recrystallization inhibition.^40^ Still, explicit evidence supporting IBP activity at low subzero temperatures and in the presence of CPAs is lacking.

This study investigates the impact of two distinct IBP types on vitrified DMSO solutions at concentrations relevant to cryopreservation procedures. The IBPs used in our research are antifreeze proteins (AFPs), which are a subset of IBPs that particularly act to depress ice growth and recrystallization.^18^ Our observations using cryomicroscopy demonstrate that AFPs impede ice growth during the warming of vitrified samples at −80 °C. Also, in the cases where ice formation did occur, the AFPs significantly depressed ice recrystallization at −50 °C. Our results unequivocally establish the activity of at least two different types of IBPs at cryogenic temperatures.

## Materials and Methods

### Materials

All materials were purchased from Merck (formerly Sigma–Aldrich) unless stated otherwise. The plasmid containing a type III AFP, QAE m1.1 isoform from ocean pout *Macrozoarces americanus*(41) was generously donated by Peter L. Davies (Queen’s University, Canada). The plasmid containing MBP-*Tm*AFP, a maltose-binding protein conjugated to the AFP from the *Tenebrio molitor* larvae,^42^ was obtained from the laboratory of Deborah Fass (The Weizmann Institute, Israel).

### AFP Production

Protein production, purification, and activity measurements are described in detail in the Supporting Information. Briefly, Type III AFP was grown in the BL21-DE3-PlysS *E. coli* strain (Novagen), and MBP-*Tm*AFP was grown in the Origami-B *E. coli* strain (Novagen). Frozen stocks were used to inoculate 100 mL of Luria broth (LB) agar (Difco, BD, France) supplemented with 100 μg mL^–1^ ampicillin. In the case of the Origami-B strain, chloramphenicol (34 μg mL^–1^) was also added. Cultures were grown at 37 °C overnight with shaking at 200 rpm. The culture was used to inoculate TB medium for fed-batch fermentation under controlled physical and chemical parameters (temperature, airflow, oxygen flow, agitation speed, foam formation, pH, and dissolved oxygen). The feed and medium components (detailed in Table S1) were supplied by direct injection into the vessel. Air supply at a rate of 0.15–2 vvm (volume of air per volume of medium per minute) maintained the dissolved oxygen concentration at >20% saturation. A pH of 7 was maintained by titration of either 2 M H_2_SO_4_ or 3 M NaOH. After 20–24 h of cultivation at 37 °C, bacterial cells were pelleted by centrifugation at 3000*g* for 45 min at 4 °C (SLA-1500, Sorvall) and stored at −80 °C until purification. Bacteria were lysed by sonication, and proteins were purified by Ni-NTA affinity chromatography or the falling water ice purification (FWIP) method,^43^ as described in the Supporting Information. Purification efficiency is presented in Figure S1. Protein activity was analyzed by thermal hysteresis (TH) measurements using a nanoliter osmometer.^44,45^

## Vitrification Experiments

### Sample Preparation

We prepared a stock solution of 80% DMSO in 20% PBS (v/v). All DMSO solutions were prepared by diluting the stock with PBS. Therefore, the DMSO solutions are buffered with PBS, and the concentrations are in v/v. To prepare the samples with 100 μM protein (AFP III, MBP-*Tm*AFP, or BSA), concentrated protein solutions were added to the 40% DMSO/PBS solution. The added volume was negligible in the final sample. The concentrated sample was diluted in 40% DMSO/PBS as needed to prepare samples with lower protein concentrations.

### Cryomicroscopy

Vitrification experiments were conducted on a Linkam MDBCS196 cold stage mounted on a microscope (Model BX41, Olympus, Japan). The system was controlled by a T95-Linkam controller equipped with an LNP95-liquid nitrogen cooling pump (Linkam Scientific Instruments, Ltd., U.K.), as demonstrated in Figure S2. We used the G7MTB sample carrier, designed to rapidly transfer flat samples from a warm position on the stage to a precooled silver block. This design allows abrupt freezing (a cooling rate of 5000 °C min^–1^ is stated by the manufacturer) with controlled preprogramming of the temperature profile under a microscope observation. A 7 mm circular quartz window placed on the sample carrier was used to reduce temperature gradients within the sample. A sample of 0.4 μL was loaded on the quartz window and covered with a 5 mm circular coverslip (5 mm diameter) to flatten the sample. The sample was sealed with immersion oil to prevent evaporation, as demonstrated in Figure S3. The silver block was precooled to −190 °C before transferring the sample from the carrier to the holder. Then, the following temperature profile was used: 3 min at −190 °C, heat to −80 °C at a rate of 10 °C min^–1^, hold at −80 °C for 10 min, heat to −50 °C at a rate of 10 °C min^–1^, hold at −50 °C for 10 min, heat to 0 °C. Real-time brightfield images were taken every 5 s using 10× and 50× magnification objectives and a QImaging EXi Aqua digital camera (QImaging, Canada).

### Image Analysis

In optical microscopy, the transmitted light from vitrified samples is indistinguishable from that from liquid water. However, the dendritic pattern of fast ice growth leads to the trapping of air bubbles within the ice. These air bubbles are detected, as demonstrated in Figure S4. We determined vitrification according to the lack of appearance of ice crystals during the cooling procedure. Experiments that had ice during cooling were omitted from all analyses.

Images of the vitrified samples were analyzed using the ImageJ 1.53c Fiji program (public domain software), as described in the SI and demonstrated in Figure S5. All statistical analysis was performed in the public domain astatsa.com. Data were analyzed with a one-way analysis of variance (ANOVA), followed by a comparison of experimental groups with the appropriate control group, using Tukey’s HSD test or Bonferroni-Holm posthoc test. The 95% confidence interval (*p* < 0.05) was considered statistically significant.

## Results and Discussion

### Design of Experiment

**Figure 2:**
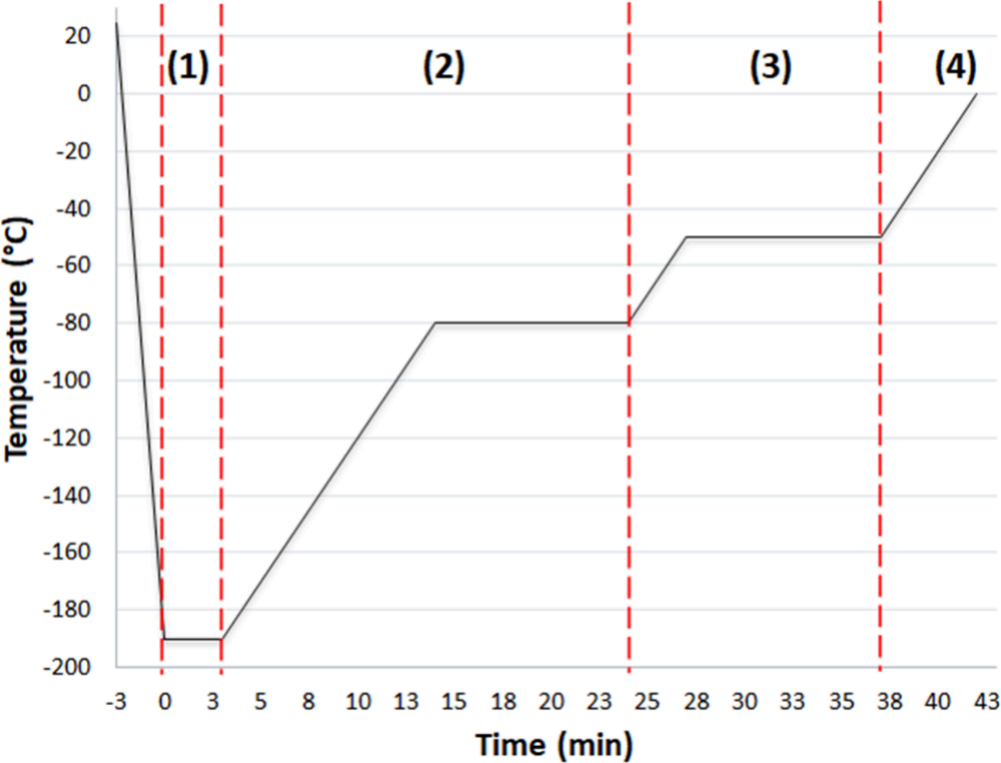
Temperature profile program used for vitrification experiments. (1) The sample is rapidly transferred onto the precooled silver block and maintained for 3 min. (2) The temperature of the silver block is raised at a rate of 10 °C min^–1^ to −80 °C, followed by incubation for 10 min. (3) The temperature of the silver block is raised at a rate of 10 °C min^–1^ to −50 °C, followed by incubation for 10 min. (4) Warming at 10 °C min^–1^ to 0 °C.

Our experimental design was tailored to mimic the temperature changes during typical vitrification procedures for the deep freezing of small biological samples. In such procedures, samples with high concentrations of CPAs undergo rapid cooling in liquid nitrogen before being transferred to −80 °C for long-term storage. For our experiments, we vitrified samples at −195 °C and subsequently subjected them to a two-step warming process with 10 min incubation periods at −80 and −50 °C (Figure 2). By conducting experiments across a range of DMSO concentrations, we gained insights into the occurrences of vitrification and devitrification within these varying concentrations. We note that the melting point of 40% DMSO solution is approximately −30 °C, as we observed experimentally and in accordance with published data.^46^ Therefore, the devitrification at −80 °C corresponds to supercooling of 50 °C. In the ice recrystallization inhibition (IRI) experiments, the sample is partially frozen, and the effective concentration of the DMSO is higher due to the freeze concentration. Therefore, the effective melting point is lower, and the measurements are conducted close to equilibrium.

### Devitrification in DMSO Solutions

#### The Vitrification Step: The Liquid-to-Glass Transition

The transition from liquid water to a glassy state for 40% (v/v) DMSO solution occurs at approximately −131 °C.^46^ Although it was not visibly discernible in our assays, we observed cracks at low temperatures below Tg in some experiments (Figure S5D), possibly due to the breaking of the stiff vitrified samples. The cracks disappeared during the warming. All of our analyses were conducted from experiments without cracks in the field of view. In cases when crystallization did occur during cooling, the presence of ice became evident through the appearance of air bubbles resulting from the rapid freezing (see Figure S5A). We, therefore, classified vitrified samples as those that did not undergo freezing during cooling. Notably, in solutions with <38% DMSO, the samples were partially frozen during cooling, indicating no full vitrification. At 40% DMSO, vitrification was observed in most of the experiments, and a higher DMSO concentration was correlated with a higher incidence of vitrification. Samples that froze during cooling were omitted from our analysis.

#### The Devitrification

**Figure 3:**
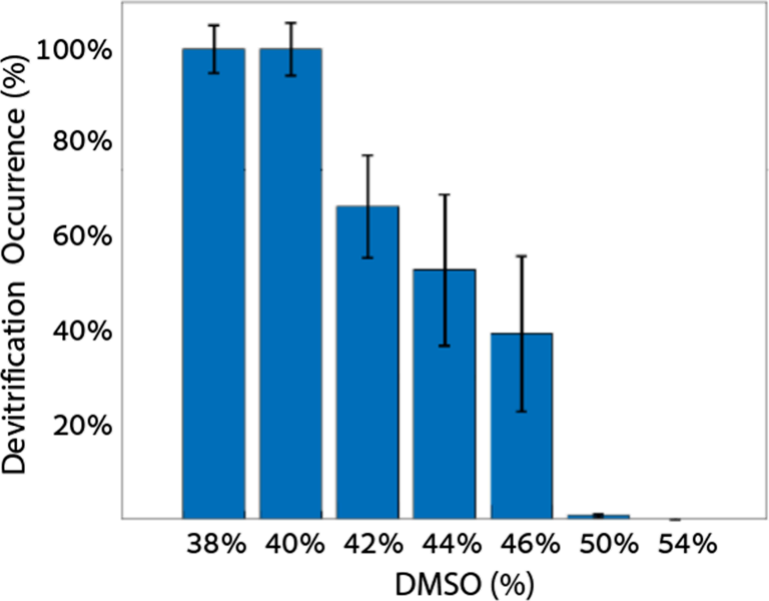
Devitrification of 38%—54% DMSO solutions at the end of incubation at −80 °C, depicted as a total gray level analysis. The data represents the mean value ± standard error of the mean (SEM). from 38% to 54%, *n* = 8, 17, 11, 8, 6, 7, and 6, respectively.

**Figure 4:**
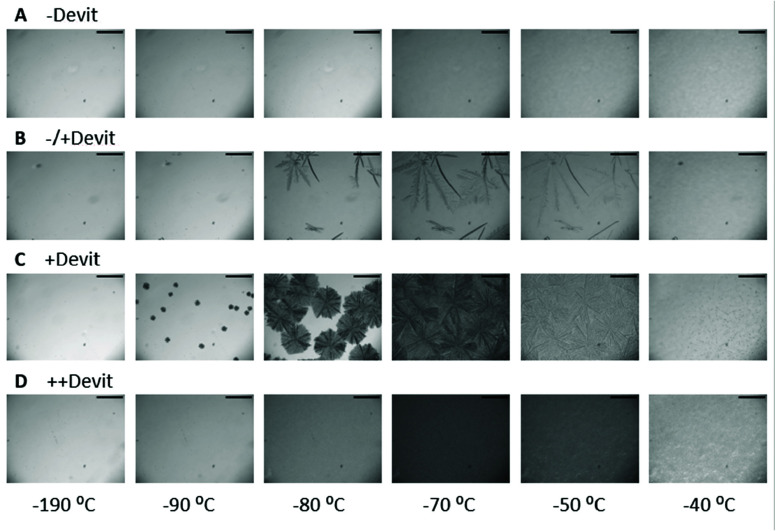
Different devitrification morphologies of DMSO solutions. Bright-field images taken during the warming of vitrified DMSO solutions from −190 at 10 °C min^–1^. The temperatures at which the images were taken are noted below. The −80 °C image was taken before the incubation period. (A) “–Devit”, which indicated that ice growth was not detected, meaning no devitrification occurred (images taken at 54% DMSO). (B) “±Devit”, where a few ice crystals indicate little devitrification (images taken at 44% DMSO). (C) “+Devit”, where ice nuclei are noted at earlier stages than “±Devit” and overall darkening appears around −80 °C to −70 °C (images taken at 42% DMSO). (D) “++Devit” which indicates the rapid growth of densely populated ice nuclei to form a bulk of ice (images taken at 40% DMSO). Scale bar = 200 μm.^49^

Ice crystals scatter light and appear dark in cryomicroscopy images (Figure 4C). As ice crystals recrystallize, they become transparent, increasing light transmission. The point in time where transmitted light is at its minimum can be considered the period of maximum devitrification prior to the dominance of recrystallization. We quantified transmitted light throughout the experiments and identified that the highest devitrification, on average, occurred at the end of the incubation step at −80 °C. This time point was used for the analysis of total devitrification. Figure 3 presents the extent of devitrification at this specific time point for all tested solutions. A higher DMSO concentration corresponds to a lower incidence of devitrification. At a DMSO concentration of 54%, we did not detect any ice formation during warming, indicating a complete absence of devitrification.

Our observations align with previous studies on vitrified samples in DMSO solutions,^46^ where devitrification was reported within 40%–50% DMSO. At lower DMSO concentrations, achieving a glass state is challenging, due to prevalent ice growth and nucleation. In contrast, devitrification is absent at DMSO concentrations of >50% . In these high DMSO concentrations, there is a significant increase in the viscosity of the solution, and the ratio of water molecules to DMSO is reduced (from a 6:1 ratio of water to DMSO molecules at 40% DMSO to a ratio of 4:1 at 50%). (See Table S3.) The increase in viscosity reduces the nucleation probability, and ice growth during warming is effectively prevented.

We note that at these high concentrations of DMSO and temperatures lower than −63 °C, DMSO hydrates may be formed. However, these hydrates were reported to be dominant for significantly higher DMSO solutions (molar ratio of 3:1 and above, equivalent to 60% (v/v) DMSO in water).^46,47^ In addition, the hexagonal shape pattern of the crystal we observed and the spicule growth patterns are characteristics of ice crystals. Recent crystallographic studies of DMSO–water hydrates show that they are monoclinic.^46,47^

#### Distinct Devitrification Morphologies

We performed a more detailed analysis of snapshots obtained during the warming phase to gain further insights into devitrification. Figure 4 illustrates the various morphologies observed. Ice crystals scatter light along their edges, making their appearance visible once their size exceeds the wavelength of transmitted light.^48^ Smaller ice crystals, considered nuclei, are below the limit detectable by cryomicroscopy. Nevertheless, when the warming rate is 10 °C min^–1^, these nuclei or crystals have sufficient time to grow or recrystallize, scattering light in the process. When multiple tiny crystals form or grow during devitrification, they become indistinguishable, leading to an overall darkening of the sample at approximately −100 °C. This phenomenon was previously termed “opacification”.^48^ In our analyses, we refer to it as “++Devit”, indicating significant devitrification. Subsequently, the ice recrystallized and melted at −40 °C.

**Figure 5:**
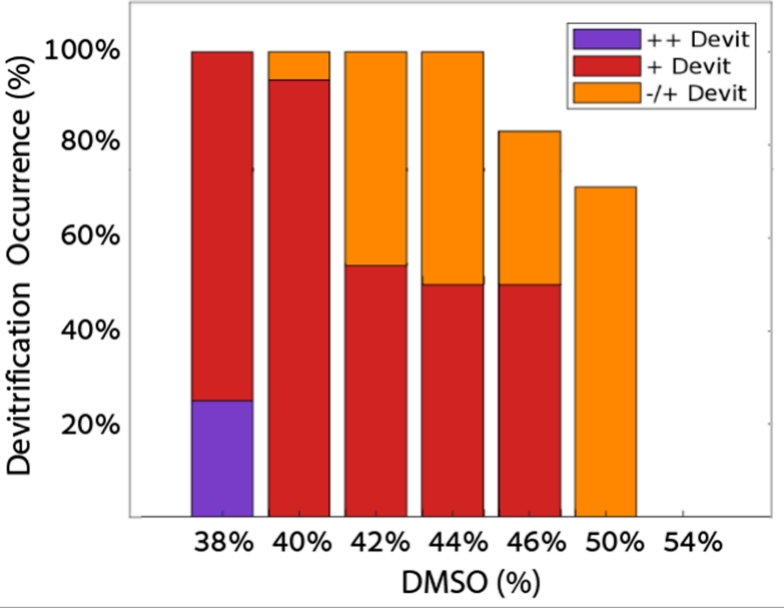
Devitrification phenomena incidence in DMSO solutions. Number of experiments in each DMSO concentration: 8 (38%), 17 (40%), 13 (42%), 8 (44%), 6 (46%), 7 (50%), and 6 (54%).

The second morphology we observed was the appearance of a few discrete ice crystals that eventually grew to fill the whole sample, with darkening of the sample at later stages of warming relative to “++Devit”. We referred to this morphology as “+Devit”. Further warming to −40 °C led to the IR and melting. The third scenario, termed “±Devit”, involved the appearance of single ice crystals without the overall darkening of the sample, suggesting fewer ice nuclei and lower ice content compared to “+Devit” and “++Devit”. The fourth category “–Devit”, signified the absence of detectable ice growth throughout warming, indicating no devitrification. As shown in Figure 5, an increase in DMSO concentration correlated with reducing the more severe devitrification occurrences of “++Devit” and an increase of the “∓Devit”. Beyond 50% DMSO, only cases of “∓Devit” were observed, and at concentrations exceeding 54%, we did not detect devitrification at all.

Fahy et al. classified vitrification into unstable, metastable, and stable.^5^ Stable vitrification, where ice nuclei do not form during cooling or warming, thereby preventing devitrification, is evident at DMSO concentrations exceeding 50%. Metastable vitrification, in which some ice nuclei form during warming, can be harmful if not properly inhibited. The “±Devit” and “+Devit” categories fall within this classification. Unstable vitrification, where multiple, undetectable ice nuclei are present during cooling, results in uniform darkening during warming, as observed in the “++Devit” category. This observation underscores the challenges of ice formation and devitrification, particularly at lower DMSO concentrations, where vitrification is less stable.

#### Influence of AFPs and BSA on Devitrification

Following our devitrification analysis of DMSO/PBS solutions, a series of experiments were conducted to evaluate the impact of AFPs on solutions containing 40% DMSO in PBS. Two distinct types of AFPs were employed in these investigations. The first type was Type III AFP, originating from the ocean pout, a protein of small molecular weight (8 kDa), compact structure, and remarkable stability. This protein can be efficiently produced in substantial quantities via recombinant techniques. Extensive research spanning over three decades has led to a thorough understanding of its structure and activity, as documented in numerous references.^18^ Nanoliter osmometer experiments revealed that Type III AFP can depress the freezing point of individual ice crystals by as much as 1 °C at millimolar concentrations.^41^ Additionally, capillary assays indicated its IR inhibition activity at concentrations as low as 200 nM.^50,51^ Fluorescent studies further revealed its preference for binding to the prism planes of ice and the irreversible binding pattern,^52^ exhibiting rapid binding kinetics relative to other AFPs.^53^

The second AFP employed in our study is the hyperactive antifreeze from *Tenebrio molitor* larvae (*Tm*AFP). In this work, a chimera of *Tm*AFP with Maltose-Binding Protein (MBP) was used to facilitate its expression in *E. coli*, as well as its detection and purification.^42^ This chimera boasted a larger size of 53 kDa, seven times that of Type III AFP. In contrast to Type III AFP, which lacks repetitive structure or sequence, *Tm*AFP features a highly repetitive beta-helix with seven repeating units, forming a nearly perfect array of exposed hydroxyl groups on its surface.^54^*Tm*AFP displays the ability to depress the freezing point of individual ice crystals by several degrees at micromolar concentrations, an effect of several orders of magnitude greater than that observed for Type III AFP.^55^ However, its IR inhibition activity is equivalent^51^ or lower than that of Type III AFP,^56^ and its binding kinetics are orders of magnitude slower.^53^

**Figure 6:**
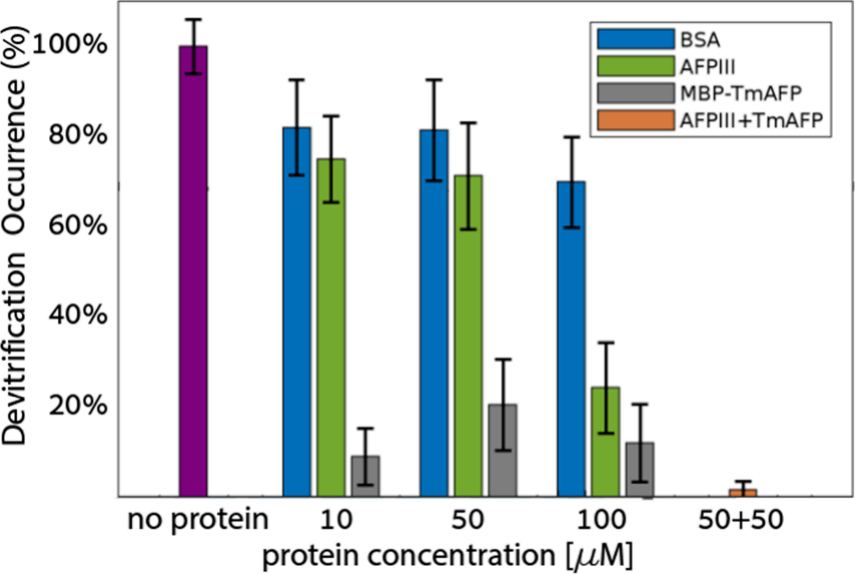
Devitrification inhibition by AFPs at 40% DMSO solutions, as depicted in a total gray-level analysis. The data represent the mean value ± standard error of the mean (SEM), *N* ≥ 14 in all experiments. The data were analyzed with a one-way analysis of variance (ANOVA), followed by a comparison of experimental groups with the appropriate control group (Tukey’s post hoc test). (*) *p* < 0.01, compared to the value of no protein and BSA at equimolar concentrations.^49^

Figure 6 presents an analysis of the darkening of samples containing 10, 50, or 100 μM AFPs in 40% DMSO solutions at the end of the incubation step at −80 °C. As discussed above, this analysis demonstrates the overall extent of devitrification of the samples. Bovine serum albumin (BSA) was employed as a control at matching concentrations. We found that, under the experimental conditions, BSA inhibits devitrification by ∼19% at concentrations of 10 and 50 μM. Type III AFP demonstrated a little more devitrification inhibition, ranging from 26% to 29% at the same concentrations. In contrast, MBP-*Tm*AFP exhibited a remarkable inhibition of 80% to 91%. A clear distinction emerged at a concentration of 100 μM, with BSA achieving 30% average devitrification inhibition, Type III AFP achieving 76%, and MBP-*Tm*AFP reaching 88%. The combination of 50 μM of each AFP type yielded a nearly complete inhibition of devitrification, with a remarkable 98.4% reduction in darkening, compared to the control case of 40% DMSO without added protein.

**Figure 7:**
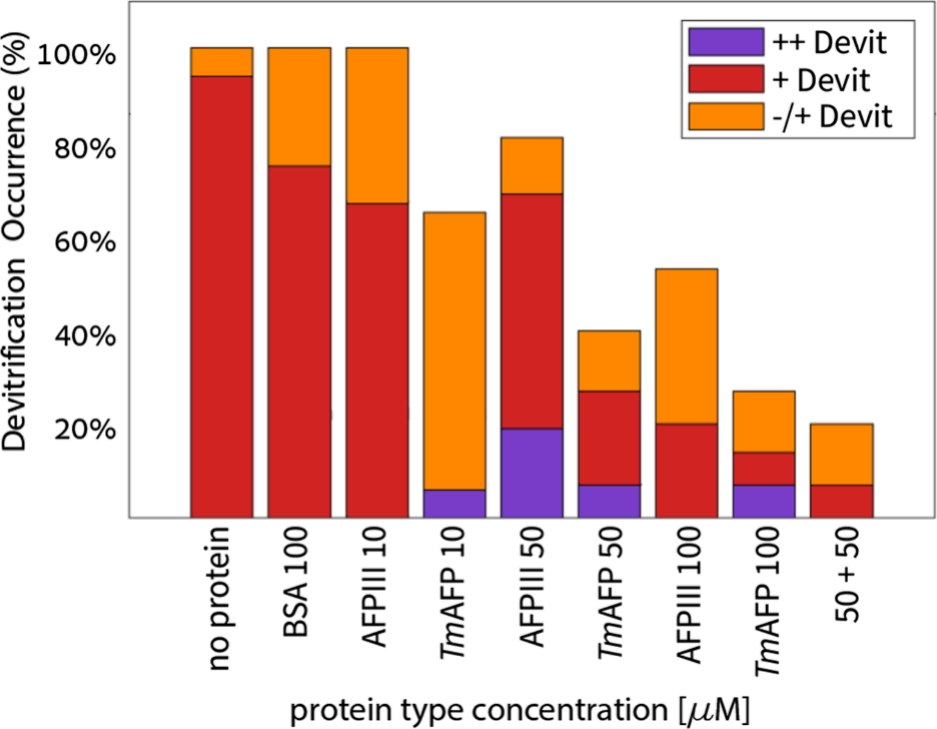
The effect of AFPs on devitrification morphologies at 40% DMSO. The devitrification morphologies are color-coded. BSA was used as the control. *n* = 15–17 for all experiments.

A more detailed understanding of the effects of the proteins on devitrification emerged from morphological analyses, as depicted in Figure 7. For BSA, it was evident that all vitrified samples experienced some level of devitrification, and no instances of complete devitrification were noted. While in the sample without protein, 94% of the cases were “+Devit” morphology and 6% were “∓Devit”, the addition of 100 μM BSA led to 75% “+Devit” and 25% “∓Devit”. Both the “+Devit” and “∓Devit” morphologies are predominantly aligned with the category of “metastable vitrification”, suggesting that the effect of BSA is not necessarily significant.

Similar results were observed for samples containing 10 μM Type III AFP. In contrast, higher concentrations of Type III AFP, and MBP-*Tm*AFP at all concentrations tested displayed significant suppression of devitrification. At a concentration of 100 μM, devitrification was inhibited entirely in 47% of the experiments with Type III AFP and 73% of the experiments with MBP-*Tm*AFP. Notably, both AFPs, while effectively reducing devitrification, led to a minor increase in the occurrence of the “++Devit” morphology, indicative of “unstable vitrification”. This was not observed with the controls, either with BSA or without protein. This finding raises the possibility that the IBPs have some ice nucleation activity under the conditions tested herein. This subject is discussed below. When the two proteins were combined, despite the overall 98% reduction in darkening, morphological analysis revealed that devitrification was inhibited entirely in 80% of the experiments. The remaining 20% of the cases included “±Devit” and “+Devit” states. For comparison, the complete depression of devitrification of each of the proteins separately at 50 μM sums up to 79% (60% for MBP-*Tm*AFP + 19% for Type III AFP). This indicates no direct synergistic effects on the proteins.

To determine whether devitrification inhibition is due to the nonspecific activity of proteins, 40% DMSO solutions were supplemented with BSA, at a molar concentration equivalent to AFP. The addition of BSA at all examined concentrations did not affect the devitrification incidence significantly. The addition of 100 μM BSA ceased to produce ice-free warming. In the samples supplemented with 10 and 50 μM BSA, the devitrification inhibition was very low, 7% and 13%, respectively. This indicates that the potent devitrification inhibition observed in AFP supplemented samples is attributed to the AFP-specific activity and not to the addition of protein.

#### Recrystallization Inhibition

**Figure 8:**
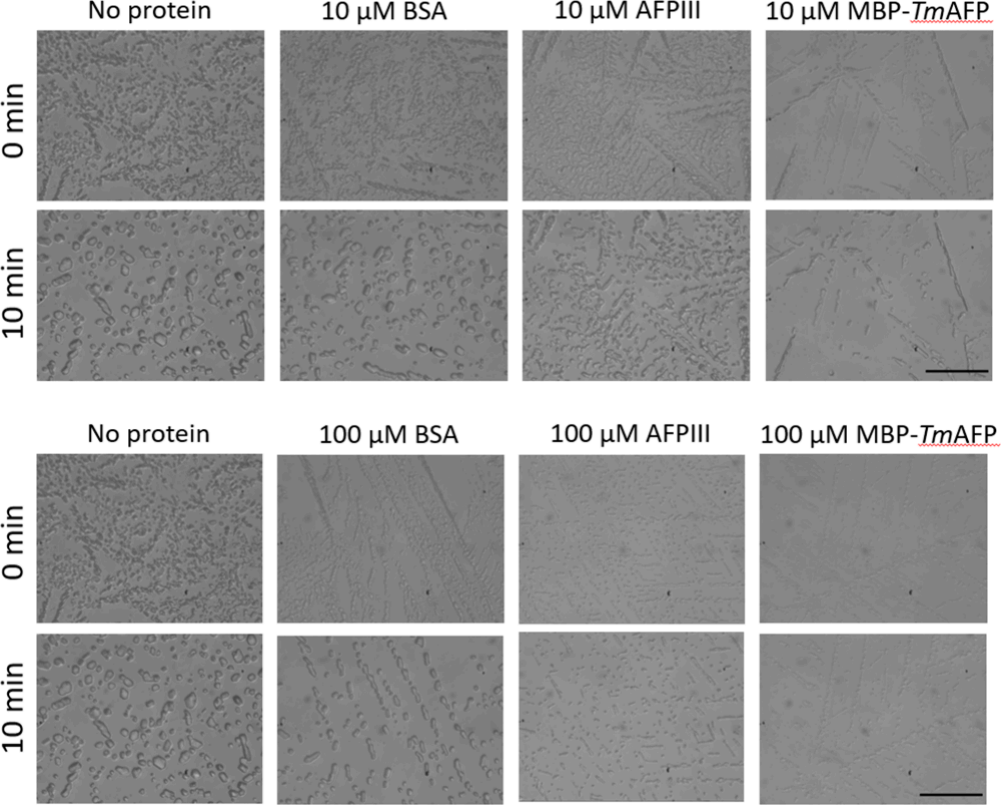
Ice recrystallization inhibition of vitrified AFP in 40% DMSO solutions at −50 °C. Scale bar = 50 μM.^49^

The phenomenon of IR necessitates the simultaneous growth and melting of ice within a system maintained at a constant temperature. As a consequence, IR occurs only when existing ice crystals are present and the diffusion rate of water is sufficiently high to enable the translation of water molecules at the surfaces of ice crystals. At vitrification temperatures, the ice crystallization process is hindered by high viscosity, and the rate of IR increases during the warming stage as temperatures approach the melting point. Our study monitored the IR of devitrified samples in the presence and absence of AFPs at concentrations of 10, 50, and 100 μM. Figure 8 and Table S2 illustrate typical transmission images of these samples at the beginning and the end of a 10 min incubation period at −50 °C.

Our findings indicate that both Type III AFP and MBP-*Tm*AFP exhibited a consistent inhibition of IR across all tested concentrations. In the case of Type III AFP, IR was entirely undetectable at all concentrations (*n* = 3 for 100 μM, *n* = 11 for 50 μM, and *n* = 12 for 10 μM). With MBP-*Tm*AFP, we observed partial IR inhibition in two of three experiments at 10 μM. At higher concentrations, IR was inhibited entirely (*n* = 2 for 100 μM, *n* = 4 for 50 μM). For comparison, BSA did not exhibit any IR inhibition in any of the experiments (*n* = 8 for 100 μM, *n* = 12 for 50 μM, and *n* = 12 for 10 μM), nor did samples without the presence of any protein (*n* = 16). It is essential to note that, in our analysis, we excluded instances of more advanced devitrification marked as “++Devit” due to the significant nucleation of ice crystals. In these cases, scattered light led to a uniform darkening of the sample, rendering it impossible to distinguish between IR and melting.

#### AFPs Activity under Cryogenic Conditions

Previous experiments with AFPs at cryogenic conditions focused on the effects of AFPs on the viability of cells and tissues after cryopreservation.^24−37^ While many studies reported positive effects, the activity of the proteins in the complex surroundings of cells remains elusive. Halwani et al. showed that the addition of a series of AFPs from *Dendroides canadensis* (DAFPs), which are homologous to *Tm*AFP, reduces the nucleation temperature of 1 M DMSO solution by 9 °C. The study also demonstrated a concentration-dependent improvement of cell viability after thawing when DAFPs were added.^24^ In accordance with those results, our findings demonstrate that AFPs in solutions of 40% DMSO depress the occurrence of devitrification at −80 °C, and inhibit IR at −50 °C, thus extending the known temperature range of the activity of AFPs. Yet, the understanding of the physics of cryopreservation systems is insufficient, even in the relatively simple case in which no cells or membranes are involved. In addition, the behavior of AFPs in the vitrified state is largely unknown. Within these limits, we discuss some optional explanations for the observed results and aspects to consider in order to understand the interactions of AFPs with ice under conditions relevant to cryopreservation.

#### The Mechanism of AFPs Activity

AFPs depress ice crystal growth and recrystallization by the adsorption–inhibition mechanism, originally suggested at the late 1970s^57^ and later on elaborated by many theoretical and experimental studies.^18,58−62^ This model mechanism suggests that the AFP molecules directly attach to the surfaces of ice crystals and block the incorporation of water molecules from the solution at the bound surfaces. The growing ice front is forced to grow in convex zones between the bound protein molecules, creating a curved interface. Consequently, the ice growth is thermodynamically less favorable due to the Gibbs–Thomson–Herring (Kelvin) effect, which leads to a noncolligative depression of the freezing point. Therefore, ice crystals in AFP solutions are stable and do not grow for a certain range of temperatures below their melting point. The adsorption is irreversible^52,62^ and the freezing hysteresis is dependent on protein type and concentration.^18^ A small hysteresis effect is also apparent during melting, and ice crystals in AFP solutions can be slightly superheated above their Tm.^63−65^

The adsorption–inhibition mechanism of ice-binding by IBPs was developed and demonstrated on ice crystals of tens of micrometers size or bigger and at temperatures close to the Tm.^18,66^ The principles behind this model, as well as other models suggested for IBPs activity, are based on the physics at the ice-water interface, where the diffusion of liquid water and protein molecules is fast in the moderately supercooling range, well above the vitrification transition temperature. The models are based on aqueous solutions without significant quantities of another solvent. In contrast, our experimental conditions include high DMSO concentrations (40% (v/v), equivalent to 5.6 M). The thermodynamic parameters of the ice and its growth morphologies in our system differ from pure ice.^67^ The temperatures in our experiments are significantly below the melting point of pure ice (0 °C) and 40% DMSO solution (−30 °C). At −80 °C, the viscosity is high; consequenly, the diffusion rate is low. In addition, many of the water molecules are replaced with DMSO molecules (see Table S3 in the Supporting Information). It is unclear that the model mechanisms for the interactions of AFPs and ice apply under these conditions. The high occurrences of devitrification in 40% (v/v) DMSO, demonstrated in Figures 3 and 4, suggests that the vitrified samples at −80 °C are in metastable glass state. Under these conditions, they are likely nucleated with nanometric ice nuclei formed during cooling or warming. The viscosity of a 40% DMSO solution increases by two orders of magnitude when the temperature drops from −30 °C to −50 °C.^68^ Close to Tg, reduction of 10 °C increases the viscosity by 3 orders of magnitude.^5^ The diffusion reverses the correlation with the viscosity. The diffusion rate may be too low in the temperature regime between Tg and Td, to allow AFP molecules to translate and rotate sufficiently fast to bind the ice nuclei and depress their growth. Compensation for the slow diffusion rates may arise from the slow ice growth rates in these temperatures and the small number of AFP molecules required to protect ice nuclei of only a few nanometers fully. In accordance with the latter assumption, fluorescence studies have established that the distance between adjacent AFP III molecules on an ice surface ranges from 10 nm to 20 nm, depending on the protein concentration.^69^ This indicates that only a minimal number of AFP molecules is necessary to protect each nucleus effectively. Although the proximity of these molecules theoretically permits supercooling by several tens of degrees without triggering ice growth, the actual TH observed in solution without high concentrations of DMSO was significantly lower. This difference is attributed to the maximum permissible contact angle between the protein and the ice–water interface, which cannot exceed 2°, relative to the protein surface.^70^ As a result, the degree of supercooling observed is less than initially expected. Nevertheless, the potential for inhibiting the growth of nanometer ice crystals at low temperatures remains substantial. In cases where ice crystal nucleation occurs near Tg, elevated AFP concentrations can further suppress the expansion of nanometer-scale ice crystals. Through interactions with AFPs, these tiny ice crystals are shielded from growth during the warming process, effectively circumventing the ice growth zone and ultimately melting.

#### Perspective on Ice Nucleation Effects of AFPs

Another optional explanation for the effect of AFPs on devitrification is the inhibition of ice nucleation. Some AFPs were shown to inhibit ice nucleators at the temperature range of heterogeneous nucleation, between approximately −7 °C to −20 °C, an effect significantly enhanced by small molecule CPAs.^71^ Yet, other works,^20−23^ including our previous work that focused on the same proteins used in the current study,^19^ showed that AFPs may promote ice nucleation in supercooled solutions in a temperature range close to the homogeneous nucleation temperature, at −35 °C to −40 °C. The effects of AFPs on heterogeneous nucleation may differ from their effect at the homogeneous nucleation regime. As shown in Figure 1, at these temperatures, the growth rates of ice are high. In contrast, ice growth rates below −50 °C are low due to the low diffusion rate of water, but the nucleation rate is high. Nucleation occurs due to local reorientation of adjacent water molecules and not due to diffusion. In our devitrification experiments, ice nucleation may occur during cooling and warming. Ice nuclei are smaller than optical microscope resolution and cannot be discriminated against from ice growth. We, therefore, cannot conclude whether the major effect of AFPs on vitrified solutions is related to nucleation inhibition or not.

Noteworthy, observing a slight increase in the “++Devit” cases in the presence of both AFPs relative to the controls implies an effect of promoting ice nucleation. Local orientation of the AFP molecules next to each other or minor aggregation of the proteins may lead to some effect of promoting ice nucleation. Qiu et al. simulated the correlation between the size of an ice-binding face and the elevation of a homogeneous freezing point. They concluded that *Tm*AFP is capable of elevating the homogeneous nucleation point by 2 °C. Also, they showed that aggregation of few *Tm*AFP molecules at the right orientation could lead to a significant nucleation effect.^22^ Clearly, the likelihood of perfect alignment of the ice-binding faces of adjacent protein molecules is small, yet this may explain the small increase in “++Devit” cases for *Tm*AFP. Since the ice-binding face of *Tm*AFP is larger than that of AFP III,^18,51,72^ it is likely that *Tm*AFP is a more effective ice nucleator relative to AFP III, as indeed measured by Eickhof et al.^19^ We note that these simulations, as well as related experimental results, refer to water without high concentrations of solute in general, and DMSO in particular. Still, these results are in accordance with our observations.

#### Nonspecific Effects of BSA on Devitrification

An interesting observation in Figures 6 and 7 is that BSA has some minor effect on devitrification compared to buffer control. Although the activity of 100 μM BSA is lower than both AFP studies at 10 μM, it is still higher than the activity measured in the buffer. BSA had no detectable effect in the IRI experiments at −50 °C. As discussed above, at −80 °C, ice nucleation governs while ice growth is not significant. Under the conditions of our experiments, ice nuclei may have formed during cooling or warming and BSA may serve as a nonspecific depressant of ice nucleation. Bonshteyn and Steponkus note that BSA slightly influences nucleation of ice in ethylene glycol solutions, and attribute it to effective elevation of the ethylene glycol concentration.^73^ Still, their BSA concentration (6%) was significantly higher than that in our experiments (0.7%).

The IRI activity of BSA, as well as other serum albumins, was previously reported. The authors suggested that the detected IRI activity may be related to the distribution of amino acids on the surface of the proteins, exposing discrete hydrophilic and hydrophobic regions. The presence of such regions may resemble, to a certain extent the charge distributions on the surfaces of AFPs, which render their affinity to ice.^74^ As mentioned, we did not observe IRI effects for BSA.

#### Effects of DMSO on AFPs and the Vitrified State

DMSO in the high concentrations used in our study affects the bulk state and may affect the protein structure. As a “supercharging” reagent, DMSO impacts the charge distribution and stability of proteins.^75^ While some works show that low DMSO concentrations can reduce binding affinities of protein–ligand complexes, in other cases, DMSO stabilizes the protein structure, even at high concentrations. For example, the presence of 40% DMSO protected the heme group at its native position in the monomeric cytochrome P450 and protected the protein from unfolding.^76^ Another study of the effect of DMSO on hen egg white lysozyme showed that the addition of up to ∼70% DMSO led to the compactization of the protein associated with increased flexibility. Unfolding occurred only above 70% DMSO.^77^ For lysozyme and myoglobin, significant unfolding occurred only at 43% and 63% DMSO, respectively.^75^ The structure of *Tm*AFP is highly stable and compact, stabilized by a series of disulfide bridges that interconnect the strands of the β sheet that form the ice-binding face of the protein. This structure proved to be highly stable at conditions of high temperature and denaturing agents.^18^ Type III AFP was also shown to be highly stable at room temperature and active at extreme pH conditions, indicating its robustness.^41^ As such, it is likely that these AFPs will not be significantly altered by the addition of DMSO.

## Conclusions

AFPs evolved in organisms as a means of adaptation to cold climates. Type III AFP is active at moderate supercooling temperatures around −1.0 °C, keeping Antarctic fish unfrozen in sea ice.^78,79^*Tm*AFP is expressed in the mealworm larvae and helps survive cold climates.^18^ In this study, we present data that show explicitly the activity of AFPs at temperatures below −50 °C, down to −80 °C (at supercooling up to 50 °C). Our findings extend the known temperature range in which AFPs are active. Consistent with this notion, an understanding of the mechanism of AFPs in the range of temperatures where diffusion is low and nucleation is high remains obscure. More research is needed to unravel the extent of the impact of AFPs under cryogenic conditions and to suggest a reliable mechanism of AFP activity at sub-biological temperatures. The results, in addition to recent improvements in large-scale production of AFPs,^43,80^ raise the potential of the addition of AFPs to cryopreservation cocktails.^7^

## Supporting Information Available

Methods [large-scale recombinant expression of IBP, protein purification and analysis, devitrification system, and image analysis–calculation of total devitrification; includes Table S1 (fermentation medium and feed composition), Figure S1 (SDS-PAGE analysis of type III AFP purification), Figure S2 (Linkam cryomicroscopy system), Figure S3 (schematic representation of sample preparation steps), and Figure S4 (image analysis of devitrification)]; results [including Figure S5 (bright-field images of liquid, crystalline, and vitrified state) and Table S2 (ice recrystallization in 40% DMSO solutions)]; discussion [including Table S3 (number of water molecules per DMSO molecule)] PDF)
