# Supplementary material for: Extended Temperature Range of the Ice-Binding Protein Activity
Source: Langmuir. 2024 Mar 25;40(14):7395–404. doi: 10.1021/acs.langmuir.3c03710 (PMC11008235; doi:10.1021/acs.langmuir.3c03710)
Supplement: Supplementary file 1 — la3c03710_si_001.pdf [file la3c03710_si_001.pdf]

Extended temperature range of the ice-binding protein activity

Supporting Information

Vera Sirotinskaya[1], Maya Bar Dolev[1,2], Victor Yashunsky[1,3], Liat Bahari[1], Ido Braslavsky[1*]

[1] Institute of Biochemistry, Food Science, and Nutrition, Robert H. Smith Faculty of Agriculture, Food and Environment, The Hebrew University of Jerusalem, Rehovot 7610001, Israel.
[2] Faculty of Biotechnology and Food Engineering, Technion, Haifa 3200003, Israel
[3] The Swiss Institute for Dryland Environmental and Energy Research, Ben Gurion University, Beer-Sheva 84105, Israel

* *ido.braslavsky@mail.huji.ac.il*

Table of Contents

A. Methods

1. **Large-scale recombinant expression of IBP** **page 2**
   Bioreactor specifications
   Preparation for operation
   Inoculum culture
   Fed-batch fermentation
   Table S1 - Fermentation medium and feed composition

2. **Protein purification and analysis** **page 3**
   Ni-NTA affinity chromatography
   Falling water ice purification
   SDS-PAGE
   Figure S1: SDS-PAGE analysis of type III AFP purification
   Thermal Hysteresis measurements

3. **Devitrification system** **page 5**
   Figure S2: Linkam cryomicroscopy system
   Figure S3: Schematic representation of sample preparation steps

4. **Image analysis - Calculation of total devitrification** **page 6**
   Figure S4: Image analysis of devitrification

B. Results **page 8**
   Figure S5: Brightfield images of liquid, crystalline, and vitrified state
   Table S2: Ice recrystallization in 40% DMSO solutions

C. Discussion
   Table S3: Number of water molecule per DMSO molecules. **page 9**

A. Methods

1. Large-scale recombinant expression of IBPs

Bioreactor specifications

All bioreactor hardware was acquired from Applikon (The Netherlands), if not noted otherwise. A 1-L or 5-L stirred tank dished bottom bioreactor equipped with an agitator, PID controller, and bioconsule was used for fed-batch culture experiments. The temperature was detected by a Pt-100 sensor and controlled by either wrapping a silicone heating blanket around the vessel or by the heat exchanger inside the vessel connected to the chiller (NesLab RTE-111, USA). Dissolved oxygen (dO_2) was measured with a steam-sterilizable electrode, culture pH with steam sterilizable pH sensor, the level control connection registered foam formation. Dry, clean air or air enriched with pure oxygen was filtered (0.22 µm filter) and supplied through an air inlet pipe. Air and oxygen flow were controlled by rotameters.

Preparation for operation

Before the cultivation dO_2 electrode and pH sensor were calibrated, positioned, and fixed to the head plate of the vessel. The vessel containing the initial batch medium (Terrific broth (TB), Table 1) was steam-sterilized for 30 min at 121 °C. Other medium components (i.e., phosphate buffer (Bio-Lab, Israel), 50% v/v antifoam solution (Sigma-Aldrich, UK), 0.5 g/mL $MgSO_4$ (Merck, Germany), and the feed (30% w/v yeast extract (Difco, BD, France) solution, 50% v/v glycerol (Romical, Israel) solution) were either sterilely filtered with a 0.22 µm filter (Merck Millipore, Ireland) or steam-sterilized for 30 min at 121 °C.

Inoculum culture

The seed culture for fermentation was prepared by inoculating 100 mL LB-ampicillin (100 µg/mL) from the working cell bank aliquote and grown at 37 °C for 17 h under normal shaking conditions (200 rpm).

Table 1: Fermentation medium and feed composition. Medium composition of the initial culture medium TB and the feeding solutions supplied during the fermentation process.

Components	Initial TB medium (per L)	Feeding solution (per L)
Yeast extract	24 g	300 g
Tryptone	12 g	
Glycerol	4 g	500 ml
Antifoam	0.7 ml	500 ml
KH_2PO_4	2.31 g	
K_2HPO_4	12.54 g	
$MgSO_4$	-	500 g
H_2SO_4	-	2 mol
NaOH	-	3 mol

Fed-batch fermentation

The initial batch medium (700 mL for 1-L vessel and 3500 mL for 5-L vessel) was inoculated with the inoculum, which constituted 5% of the volume of the initial batch medium. The culture was grown during the biomass accumulation stage at 37 °C. Before the induction, the temperature was lowered to 15–25 °C (depending on the target IBP expressed) then the culture was induced with 1 mM of IPTG and cultivated at the protein production stage till harvest. The feed and medium components were supplied by direct injection into the vessel. Air or oxygen-enriched air was supplied at a rate of 0.15–2 vessel volumes per minute (vvm) to maintain the dO_2 concentration at more than 20% of saturation. The agitation rate was maintained at 200–1200 rpm. A PID controller was used to maintain the pH at 7 with the addition of either 2M H_2SO_4 (Sigma-Aldrich, UK) or 3M NaOH (Gadout-Group, Israel). pH titration and antifoam solution were supplied by the peristaltic pumps. Cells were cultivated until harvest. At the end of cultivation bacterial culture was drained from the sample outlet. Bacterial cells were pelleted by centrifugation at 3000 g for 45 min at 4 °C (SLA-1500, Sorvall) and stored at −80 °C until purification.

The cell density was determined by measuring the culture optical density (OD) at 600 nm with a spectrophotometer (Genesys 50, Thermo Scientific). A standard ratio of OD = 0.4 g DCW/L (i.e., dry cell weight per liter) was then used to convert the optical density to dry cell weight.

2. Protein Purification and analysis

The cell pellet was thawed on ice and resuspended in a lysis buffer (50 mM sodium phosphate buffer, 300 mM NaCl, pH 7.5, (Bio-Lab, Israel)) supplemented with protease inhibitors (0.57 µM AESBF, 0.05 µM Bestatin, 0.007 µM Leupeptin, and 0.007 µM Pepstatin A (Sigma-Aldrich, UK)). The resuspended pellet was lysed by sonication with an amplitude of 80% (CV33, Sonics Vibra-Cell) and centrifuged at 27000g for 45 min at 4 °C (SS-34, Sorvall). The supernatant was passed through a 0.22 µm filter (Merck-Millipore, Ireland) purified by Ni-NTA affinity chromatography or ice affinity purification.

Ni-NTA affinity chromatography

Soluble AFP (supernatant fraction) was bound to nickel-nitrilotriacetic acid (Ni-NTA) resin (Bio-Rad, Germany) for 1 h at a rotary shaker (Intelli-mixer, ELMI, USA) at 4 °C. The supernatant fraction mixed with Ni-NTA beads was transferred to the column, allowed to settle, and flow-through fraction was collected. The beads were washed with 3–4 column volumes of the lysis buffer and eluted in a step gradient of increasing imidazole (Sigma-Aldrich, UK) with elution buffer (lysis buffer supplemented with 20–200 mM imidazole) until no protein was detected by Bradford assay (Bio-Rad, Germany). AFP-containing fractions were buffer-exchanged against 1X PBS buffer (pH 7.4) in SnakeSkin dialysis tubing (Thermo Fisher Scientific, USA) 3.5 kDa MWCO for type III AFP, and in Visking dialysis tubing (Medicell Membranes Ltd, England) 10 kDa MWCO for fusion IBPs and then concentrated if needed.

Falling water ice purification

We used the Falling Water Ice Purification (FWIP) to purify large amounts of recombinant AFPs produced by fed-batch fermentation, as published. FWIP utilizes a commercial ice-making machine (KM-35A, Hoshizaki, Japan) with an ice production capacity of 36 kg ice per 24 h. The machine accommodates two metal plates with a surface area of 900 cm^2 each, on which ice grows. The ice is produced in cycles. When the cycle starts, diluted protein lysate is supplied to the machine, and it flows over the surface of the metal plates. The temperature of the plates is lowered gradually, and ice cubes grow on their surface. After the batch of ice is produced at the end of the cycle, it is defrosted and stored in the machine storage bin. Then, the residual lysate is drained away, and another cycle starts. The machine can consume 0.6 - 2.5 L of the loading solution in one cycle and produce 0.15–0.7 L of ice in 12–40 min.

We determined a dilution factor of 40x as a minimal dilution of the E. coli lysate for the FWIP procedure. The maximal concentration of the total protein (non ice-binding proteins) in the loading solution was determined to be 0.16 mg/mL. Type III AFP was purified on FWIP. 20 g (wet cell weight) of the E. coli BL21 pellet obtained from fermentation experiments were resuspended in 160 mL of 50 mM sodium phosphate buffer (pH 7.5), lysed, and centrifuged as described in section 2.1.4. The clarified supernatant of cell lysate was diluted to 9 L with double distilled water (DDW) and phosphate buffer to obtain the final salt concentration of 50 mM. The diluted cell lysate (i.e., loading solution) was cooled to 4 °C and supplied to the ice machine manually or from the pressure tank (Kspark, Taiwan). The loading solution was then allowed to circulate over the metal plates, the ice cubes grew and incorporated IBP, the buffer salts and other impurities were excluded. After the freezing cycle was completed, the ice (I1) was rinsed with cold DDW or fanned with dry air to remove the residual liquid and melted. The drain was collected, diluted with DDW to reach the buffer concentration of 50 mM, and reloaded into the
ice machine for another purification cycle. After completing a set of purification cycles (termed “round”), all the ice fractions were titrated with a phosphate buffer, concentrated, dialyzed, and assessed for purity and activity. To reach higher purity, the melted ice was loaded again for another purification round. 5 mL samples were collected from the fractions during the purification process and concentrated in Vivaspin centrifugal concentrators (Sartorius, UK) with 3 kDa MWCO to the final volume of 0.5 mL and analyzed by SDS-PAGE (Figure S1).

SDS-PAGE

The purity of fractions collected during the purification processes was assessed by SDS-PAGE (Figure S1). The optimal purification procedure eventually included two rounds of FWIP.

The final stock solutions of type III AFP and MBP-*Tm*AFP were prepared in a 1X PBS buffer (pH 7.4) and concentrated in Vivaspin centrifugal concentrators (Sartorius, UK) with 3 kDa MWCO (for type III AFP) and 10 kDa MWCO (for fusion IBPs).

SDS-PAGE and protein concentration determination SDS-PAGE was performed in a 6% stacking gel, 12 - 18% resolving gel following standard procedures. 231 Protein concentration was determined by Micro BCA Protein Assay (Thermo Scientific, USA) using

bovine serum albumin (BSA) as a standard. The absorbance was read at 550 nm using an ELx808 ultra microplate reader (BioTek Instruments, USA).

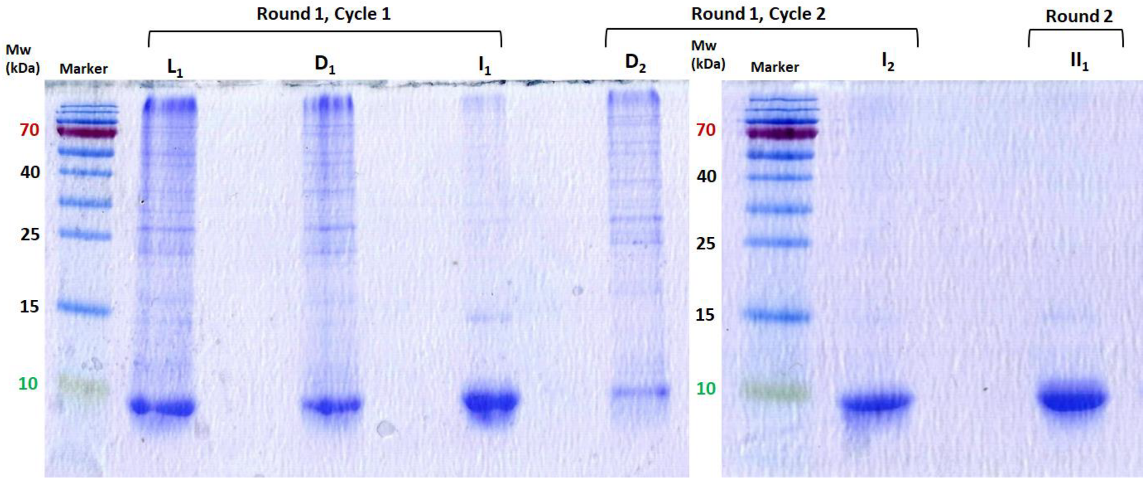

Figure S1: SDS-PAGE analysis of fractions obtained from type III AFP purification by FWIP. From left to right: marker (protein molecular weight on the left); E. coli BL21 lysate supernatant containing type III AFP, i.e., initial loading solution (L1); drain (D1) and ice (I1) fractions obtained from the first cycle of purification round 1; drain (D2) and ice (I2) fractions obtained from the second cycle of purification round 1 after fractionation of D1; ice fraction (II1) obtained from the first cycle of purification round 2 after fractionation of I1. The samples were concentrated 10-fold. The SDS-PAGE was performed in a 6% stacking gel, 15% resolving gel, then stained with Coomassie blue.

Thermal hysteresis measurements

Antifreeze activity was assessed by measuring thermal hysteresis using a LabVIEW-controlled nanoliter osmometer as described elsewhere 32. Briefly, a 10 nL sample was placed by a glass capillary in a copper disc cavity filled with immersion oil. The disc was positioned on the cold stage of a microscope (Olympus BH-2, Japan) with the controlled temperature operated by the LabVIEW software. First, the sample was rapidly cooled to −40 °C and frozen. Then the temperature was gradually increased to melt the bulk ice until a single crystal remained. The crystal was melted to the size of about 10 μm diameter. The highest temperature at which melting has ceased was determined to be the melting temperature of the crystal. Next, the temperature was set to a few hundredths of a Celsius degree below the melting point to maintain the crystal stable in the solution and exposed to AFP. Upon completion of the 10 min exposure time, the temperature was decreased automatically at a 0.01 °C each 4 s until the sudden burst of the ice crystal occurred. The TH was calculated as a difference between the crystal melting temperature and the crystal burst temperature.

3. Devitrification system

The cold stage accommodates an isolated chamber that contains a silver block, a warm post, and a sample carrier (Figure S2). The silver block is cooled by liquid nitrogen supplied by the pump and is heated by an electrical heating element. This block has a 1 mm aperture hole to allow the passage of light through the sample. The sample carrier can be moved automatically by the 2D-manipulator motor or manually by the manipulator thumbscrews.

The G7MTB sample carrier used for the vitrification experiments bears a 7 mm diameter stainless steel ring as a sample holder. A quartz window was located under the steel ring to sit firmly inside its perimeter. The sample was prepared between the quartz window and a coverslip as demonstrated in Figure S3.

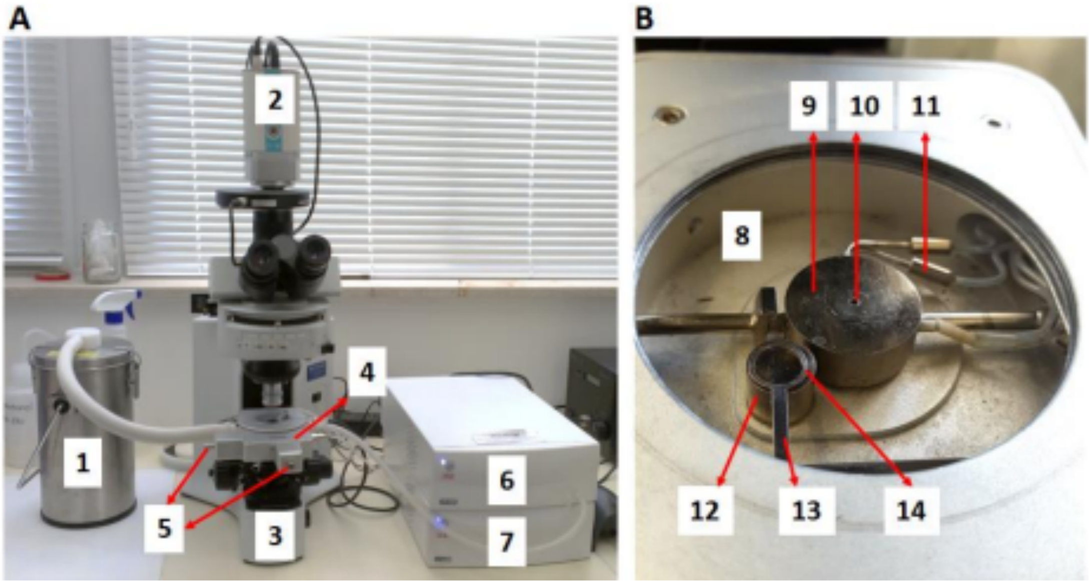

Figure S2: Linkam cryomicroscopy system used for the vitrification experiments. Images of the Linkam cryomicroscopy system (A) and the cold stage (B). 1. Liquid nitrogen dewar, 2. QImaging EXi Aqua digital camera, 3. Olympus BX41 microscope, 4. Linkam MDBCS196 cold stage, 5. Manual manipulator thumbscrews, 6. T95-Linkam system controller, 7. LNP95-Liquid nitrogen pump, 8. Cold stage chamber, 9. Silver cooling/heating block, 10. Aperture hole, 11. Heating element wire, 12. Warm post, 13. G7MTB sample carrier, 14. 7 mm diameter stainless steel ring.

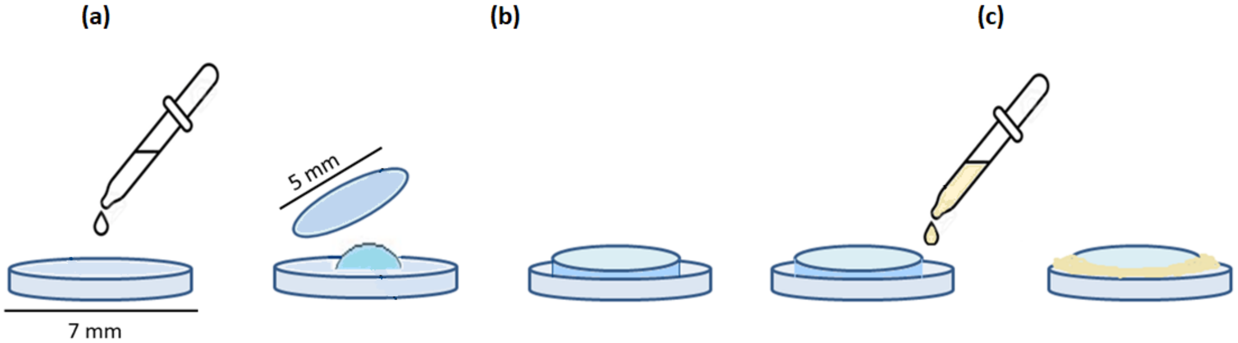

Figure S3: Schematic representation of sample preparation steps. (a) A 0.4 μL droplet of the sample solution is loaded on the quartz window. (b) The droplet is covered with a circular glass coverslip. (c) The gap between the quartz window and the coverslip is sealed by immersion oil on the perimeter.

4. Image analysis - calculation of total devitrification amount

Sequence of images comprising typically 285 images was loaded into the ImageJ Fiji software, capturing the warming process from −190 °C to −50 °C and a subsequent 10-

minute incubation at −80 °C. A custom program, employing a sequence of ImageJ commands (macro), was then executed on this image series to quantify the area of ice crystals within the stack. Specifically, the macro computed the average gray value of each image by employing a threshold to distinguish between the background (liquid) and the developing ice (Fig. S4).

The average gray value, ranging from 0 to 255, serves as an indicator, where 0 represents liquid and 255 represents ice. Based on the set threshold, each pixel in the image is assigned a gray value of either 0 or 255, and the mean gray value for all pixels is then calculated. The resulting means of the gray values across all images in the sequence are normalized by dividing by 255 and then multiplied by 100 to obtain the percentage of the sample field of view covered with ice. Therefore, a higher average gray value in an image corresponds to a larger area covered with ice.

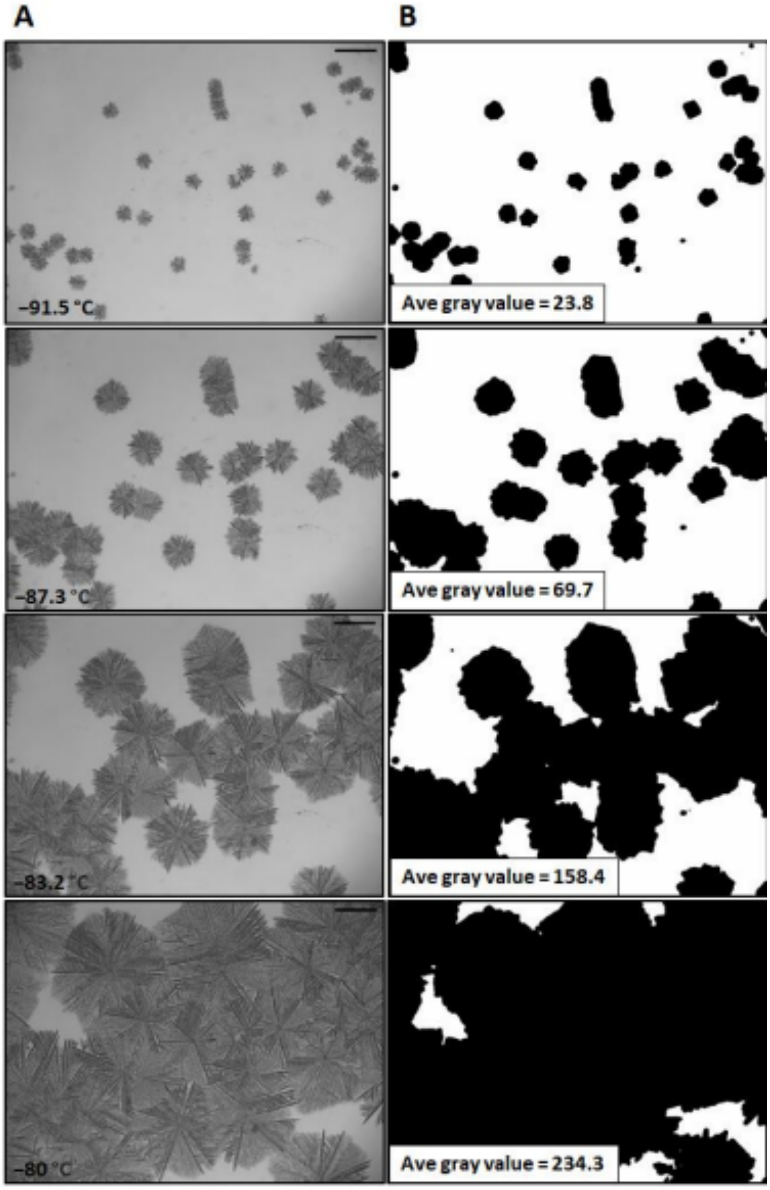

Figure S4: Image analysis of devitrification. Calculation of gray level as indication of ice growth kinetics during the warming of vitrified DMSO solutions. (A) Images of 40% DMSO solutions during the warming from −190 °C to −80 °C at 10 °C/min. (B) Same images as in (A) after the macro was applied. Scale bar 100 µm.

B. Results

Devitrification incidence in DMSO solutions

In the first place, the devitrification incidence was examined in 38%—54% v/v DMSO-PBS solutions. In this range of DMSO concentrations at the given experimental conditions, vitrification of the sample is achieved without visible crystallization when the solution is cooled. Figure S4 shows brightfield images of different phases: crystalline of double distilled water (DDW) at −190 °C, and liquid and glassy states of 40% v/v DMSO solution at 0 °C and at −190 °C that were cooled as described in the main text. DDW did not undergo vitrification, but dendritic crystallization, as indicated by the air bubbles trapped inside ice Figure S5-A). 40% DMSO solution at 0 °C was in a liquid state (Figure S5-B); at −190 °C, it was in a glassy state (Figure S5-C), though the two looks alike. 40% DMSO solution after vitrification frequently undergoes cracking, indicating a glassy state (Figure S5-D). This cracking is impossible in a liquid state and is a clear visual indication of the vitrified state.

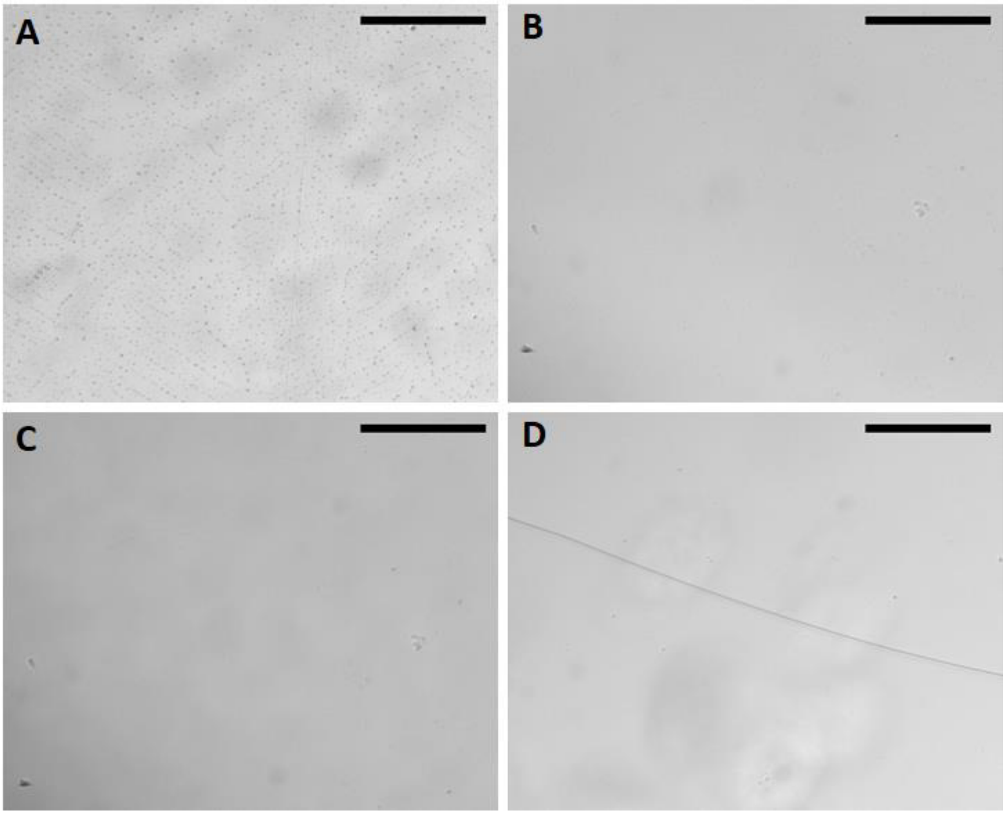

Figure S5: Brightfield images of liquid, crystalline, and vitrified state. (A) crystallized DDW at −190 °C, (B) liquid 40% DMSO solution at 0 °C, (C) vitrified 40% DMSO solution at −190 °C (D) vitrified 40% DMSO solution that underwent cracking at −190 °C. Scale bar 100 µm.

Table S2: Ice recrystallization in 40% DMSO solutions. The IR was observed during the 10 min incubation at −50 °C. “+” - visible ice recrystallization, “−” - ice recrystallization is inhibited, and “±” - ice recrystallization is partially inhibited. n is the number of experiments.

Treatment	Ice Recrystallization at −50 °C			n		
40% DMSO	+			16		
	10 µM	50 µM	100 µM	10 µM	50 µM	100 µM
40% DMSO + BSA	+	+	+	12	12	8
40% DMSO + type III AFP	−	−	−	12	11	3
40% DMSO + MBP-*Tm* AFP	±	−	−	3	4	2
40% DMSO + type III AFP + MBP-*Tm* AFP			−			1

C. Discussion

Table S3: Number of water molecules per DMSO molecules

v/v DMSO	w/w DMSO	DMSO Molarity	DMSOmol%	#H2O/#DMSO
38	40.3	5.4	13.4	6.4
40	42.3	5.6	14.5	5.9
42	44.3	5.9	15.5	5.4
44	46.4	6.2	16.6	5.0
46	48.4	6.5	17.8	4.6
48	50.4	6.8	19.0	4.3
50	52.4	7.0	20.2	3.9
52	54.4	7.3	21.5	3.6
54	56.4	7.6	22.9	3.4
56	58.3	7.9	24.4	3.1
58	60.3	8.2	25.9	2.9
60	62.3	8.4	27.5	2.6
62	64.2	8.7	29.3	2.4
64	66.2	9.0	31.1	2.2
66	68.1	9.3	33.0	2.0
70	72.0	9.9	37.2	1.7
75	76.7	10.6	43.2	1.3
80	81.5	11.3	50.3	1.0
